# Molecular docking analysis and dynamics simulation of salbutamol with the monoamine oxidase B (MAO-B) enzyme

**DOI:** 10.6026/97320630018304

**Published:** 2022-03-31

**Authors:** Hasanain Abdulhameed Odhar, Ahmed Fadhil Hashim, Suhad Sami Humad

**Affiliations:** 1 Department of pharmacy, Al-Zahrawi University College, Karbala, Iraq

**Keywords:** Salbutamol, MAO-B, Parkinson's disease, docking, molecular dynamics simulation

## Abstract

The monoamine oxidase B (MAO-B) enzyme is linked with Parkinson's disease. Therefore, it is of interest to document the molecular docking analysis and dynamics simulation of salbutamol, a well-known β2-adrenoceptor agonist, with the monoamine oxidase B (MAO-B) enzyme for further consideration in drug design and development.

## Background:

Parkinson's disease (PD) is a neurodegenerative illness that primarily influence elderly people between the age of 55 and 65 years [1]. PD is mainly characterized by the progressive damage and loss of dopaminergic nerve cells in the substantia nigra pars compacta area of midbrain. When 60% -70% of these dopaminergic neurons are lost, four motor related symptoms will emerged and these are: resting tremor, bradykinesia, muscles rigidity and postural imbalance [2,3]. As PD develop over time, non-motor manifestations like depression may also arise [4]. PD is also characterized by the buildup of Lewy bodies within dopaminergic neurons, these Lewy bodies are considered proteinaceous inclusions made up mainly of α-synuclein that is misfolded [5]. Although no precise cause of PD has been specified, several factors are reported to have positive association with development of PD like pesticides and genetic factors. On the other hand, it is believed that both smoking and consumption of coffee may lower the risk of PD [6]. The pathology of PD seems to be complex and involves numerous etiological pathways. As such, designing a drug molecule for PD treatmentthat target a single pathway appears to be ineffectual therapeutic approach. Currently, there is an urgent need to introduce a drug candidate that can target multiple pathways within PD pathogenesis network [7]. The available treatment options for PD, like levodopa and monoamine oxidase B (MAO-B) inhibitors, focus mainly on the mitigation of symptoms but with no ability to modify disease course [8,9 - check with author].

As PD disease course advances over time, both Lewy bodies and α-synuclein are increased in the brain of affected patients [10]. It is believed that targeting α-synuclein metabolism may reduce the accumulation of this protein in affected neurons and thereby halt the progression of PD disease course. In this direction, several cell line studies showed that β2-adrenoceptor agonists like salbutamol can reduce the expression of α-synuclein by modulating the acetylation of lysine 27 of histone H3. Treatment of human neuroblastoma cell model with propranolol, a non-selective β-adrenoceptor antagonist, was found to increase the concentration of α-synuclein in these cells [11].Additionally, two epidemiological studies have suggested a positive association between PD risk and administration of β-adrenoceptor antagonists. These two studies also proposed that exposure to β2-adrenoceptor agonists may decrease the risk of PD [11,12]. However, this epidemiological link between PD risk and β-adrenoceptor agonists or antagonists was abolished when association was adjusted to confounding factors like essential tremor and smoking [13]. Moreover, three small clinical trials had observed a beneficial effect when salbutamol was added to levodopa in PD patients [14-16].

The beneficial effect of salbutamol in PD patients may not be only due to α-synuclein lowering effect. Animal studies suggested that administration β-adrenoceptor agonists can enhance brain extraction of levodopa and leucine, possibly through peripheral mechanisms that decrease the concentration of other competinglarge neutral amino acids (LNAAs) for transport. Thus, the adjunct use of β-adrenoceptor agonists in PD patients may enhance delivery of levodopa to brain, reduce daily requirement of levodopa and improve clinical symptoms [17]. In this computational study, we proposed that the adjunct use of salbutamol may have an additional useful effect in PD patients treated with levodopa. We hypothesized that salbutamol may be able to selectively inhibit monoamine oxidase B (MAO-B) and thereby decreases the degradation of dopamine in central nervous system. It is well known that selective inhibition of MAO-B enzyme can decrease daily requirement of levodopa in PD patients by elevating the level of both endogenous and exogenous dopamine [18]. Also, inhibition of MAO-B can reduce oxidative stress by lowering the production of hydrogen peroxide, a byproduct of oxidative deamination reaction [19]. Molecular docking analysis had been applied by many studies to design and evaluate selective and potent MAO-B inhibitor candidates as summarized in Table 1(see PDF). The main idea of this computational study is illustrated in Figure 1(see PDF).

## Methodology:

We used molecular docking to assess the binding affinity and selectivity of salbutamol against monoamine oxidase A (MAO-A) and monoamine oxidase B (MAO-B) crystals. Then, the docking results were further validated by employing molecular dynamics (MD) simulation.

## Molecular docking:

The binding orientation and affinity of salbutamol with MAO-A and MAO-B crystals were assessed using the 1-CLICK DOCKING tool available in Mcule.com platform for drug discovery [20]. This online platform has an embedded version of AutoDock Vina to carry out docking operations [21].The two-dimensional structure of salbutamol was drawn in the board of 1-CLICK DOCKING tool. Then, docking was performed against only chain A of MAO-A (PDB: 2BXR) and MAO-B (PDB: 2BK3) crystals [22,23]. The platform of Mcule.com uses AutoDock tools to prepare both ligand and target for docking process [24]. The employed docking coordinates were (X: 21.0, Y: 2.0, Z: 6.0) for the MAO-A crystal data. We applied the following coordinates (X: 56.0, Y: 153.0, Z: 23.0)for the MAO-B crystal. The binding site area used for both MAO-A and MAO-B was (22*22*22) Angstrom.The ligand-enzyme complex with least energy of binding pose were then evaluated and visualized by using both PyMOL v2.4.1 and Discovery Studio Visualizer v21.1.0.20298 [25, 26].

## Molecular dynamics (MD) simulation:

The docking results were then further validated through molecular dynamics (MD) simulation for 50 nanoseconds. In this MD study, the docking complex between salbutamol and each enzyme with the minimum energy of binding pose was submitted for simulation by YASARA Dynamics v20.12.24 [27]. For this simulation study, the followed steps are similar to what we have used in our previous research articles [28-31 - check with author]. As a summary, the simulation includes an optimization of hydrogen bonds network with a prediction of pKa value to fine-tune amino acid residues protonation at pH 7.4 [32]. Also, sodium chloride was added in a concentration of 0.9% and an excess of sodium or chloride were applied to neutralize the complex of salbutamol and enzyme. In addition, steepest descent and simulated annealing minimizations were used for simulation to remove any possibility of clashes. The following force fields were applied during MD simulation: AMBER14 for solute, TIP3P for water, GAFF2 and AM1BCC for ligand [33-35].For this MD study, van der Waals forces cut-off value was 8 Angstrom and default parameters were used for AMBER [36]. On the other hand, no cut-off value was applied for electrostatic forces as Particle Mesh Ewald algorithm was used for this simulation [37]. At a pressure of 1 standard atmosphere (atm) and a temperature of 298K, motions equations were applied as a multiple time step of 1.25 and 2.5 femto seconds for bonded and non-bonded interactions respectively [38]. After evaluation of the root-mean-square deviation (RMSD) for solute as a function of simulation interval, the first 50 nanoseconds were considered as equilibrium time and excluded from any additional analysis. And lastly, GraphPad Prism version 8.0.2 was employed to plot and evaluate ligand movement RMSD of salbutamol throughout simulation interval.

Molecular Mechanics Poisson-Boltzmann Surface Area (MM-PBSA) binding energy was calculated for each docking complex of salbutamol and enzyme by employing AMBER14 force field. A built-in macro within YASARA Dynamics software has the capacity to carry out calculations of MM-PBSA binding energy both easily and automatically. As mentioned in YASARA guideline, the more positive binding energy refers to better interactions between ligand and target [39,40 - check with author]. The following equation was used by YASARA Dynamics to calculate binding energy:

Binding Energy= EpotRecept + EsolvRecept + EpotLigand + EsolvLigand - EpotComplex - EsolvComplex

## Results and Discussion:

According to docking results, the energy of binding for salbutamol against MAO-A and MAO-B monomers were 7.1 and 7.2 Kcal/ mol respectively. As reported here, the energy of binding for salbutamol with active site of MAO-A and MAO-B isozymes are almost equal. However, two-dimensional images for docking results in Figure 2(see PDF) showed that salbutamol can exhibit a more preferred orientation within MAO-B active site as compared to MAO-A. As seen in Figure 2(see PDF) (A), salbutamol is involved in unfavorable donor-donor interaction with Lysine 286 residue of chain A for MAO-A crystal. The formation of unfavorable interactions in docking results may indicate the presence of repulsive forces between ligand and target. Therefore, the generation of these unfavorable interactions can adversely influence the stability of ligand-target complex in docking studies [41]. Despite the fact that salbutamol docking energy against MAO-A and MAO-B are almost identical but the binding orientation of salbutamol within MAO-B active site may be more favorable due to absence of unfavorable interactions in two-dimensional docking image, as seen in Figure 2(B)(see PDF).

Docking results were then further validated by molecular dynamics (MD) simulation for 50 nanoseconds. By superposing the complex between salbutamol and enzyme on its reference structure, the proximity of salbutamol to enzyme active site was reported in Figure 3(see PDF) as a function of simulation time. As can noted in Figure 3(see PDF), salbutamol was able to maintain a closer proximity to MAO-B active site as compared to MAO-A. Throughout simulation period, the mean ligand movement RMSD for salbutamol against MAO-A and MAO-B was 9.55 and 2.94 Angstrom respectively. As such, the closer proximity of salbutamol to MAO-B active site throughout simulation time may indicate a stronger interaction as compared to MAO-A.

The calculations of MM-PBSA binding energy indicate that salbutamol may have a better interaction with MAO-B active site than MAO-A. According to YASARA guide, the more positive binding energy refers to better interaction between ligand and target. And in this regard, the reported average MM-PBSA binding energy for salbutamol against MAO-A and MAO-B was -12.09 and -6.03 Kcal/ mol respectively.So, MM-PBSA binding energy for salbutamol against MAO-B active site is more positive than that against MAO-A.

## Conclusion:

We document the molecular docking analysis and dynamics simulation of salbutamol, a known β2-adrenoceptor agonist, with the monoamine oxidase B (MAO-B) enzyme for further consideration in drug design and development.
